# Establishing action levels for EPID‐based QA for IMRT

**DOI:** 10.1120/jacmp.v9i3.2721

**Published:** 2008-06-23

**Authors:** Rebecca M. Howell, Iris P. N. Smith, Christie S. Jarrio

**Affiliations:** ^1^ Department of Radiation Physics The University of Texas M. D. Anderson Cancer Center Houston Texas U.S.A.; ^2^ Department of Radiation Oncology Emory University School of Medicine Atlanta Georgia U.S.A.

**Keywords:** Portal dosimetry, EPID, IMRT QA, gamma index

## Abstract

Although portal dosimetry is used to provide quality assurance (QA) for intensity‐modulated radiation therapy (IMRT) treatment plans, trends in agreement between the portal dose prediction (PDP) and the measured dose have not been clarified. In this work, we evaluated three scalar parameters of agreement for 152 treatment plans (1152 treatment fields): maximum gamma $\left(\gamma_{\text{max}}\right)$, average gamma $\left(\gamma_{\text{avg}}\right)$, and percentage of the field area with a gamma value greater than 1.0 $\left(\gamma_{\%}>1\right)$. These data were then used to set clinical action levels based on the institutional mean and standard deviations. We found that agreement between measured dose and PDP was improved by recalculating the fields at lower dose rates. We conclude that action levels are a useful tool for standardizing the evaluation of EPID‐based IMRT QA.

PACS numbers: 87.53.Oq, 87.53.Mr, 87.53.Xd

## I. INTRODUCTION

Different methods are used for quality assurance (QA) of intensity‐modulated radiation therapy (IMRT) fields, including the use of film and ion chamber measurements and detector arrays, such as MapCHECK.^1^, ^4^ In our clinic, we recently implemented Varian Portal Dosimetry System (Varian Medical Systems, Palo Alto, CA), which uses an electronic portal imaging device (EPID), to measure the dose from the delivery of an IMRT treatment. We based our decision (to switch to this technique for IMRT QA) on the considerable amount of research that has been published reporting that the use of EPIDs is suitable for IMRT QA.^5^, ^12^

To perform QA for IMRT with portal dosimetry, one uses analytic software to correlate the response of an amorphous silicon (aSi) EPID to the dose delivered by applying position‐specific factors from a dose calibration table. This calibration table is determined using the calibration protocol outlined in the portal dosimetry system's users' manual. Briefly, to calibrate the portal imager one must first determine the EPID response at different locations throughout the portal imager. Then, one must correlate the portal imager responses to the dose measured by a reference dosimeter, such as an ionization chamber with a calibration traceable to a National Institute of Standards and Technology (NIST) laboratory. Once calibrated, the acquired portal doses are displayed in calibration units (CU). The CU are derived by irradiating the portal imager under reference conditions (i.e., by delivering a set number of monitor units [MU] that is known to deliver a given dose), thereby relating CU to MU as well as dose. One must perform the calibration procedure for each energy and dose rate combination that will be used for IMRT and IMRT QA. In implementing our IMRT QA system, we completed the calibration protocol during its initial commissioning and quarterly thereafter. A source‐to‐imager distance (SID) of 105 cm was used for the calibration and all QA field deliveries.

During the first months we used IMRT QA with portal dosimetry in our clinic (September 2006 to December 2006), individual physicists who performed the QA analysis decided on a case‐by‐case basis if a particular result was “acceptable,” that is, whether a predicted dose was accurate to a clinically acceptable level or whether it was sufficiently inaccurate that the IMRT treatment plan was unsuitable for patient treatment. However, no action levels were predefined. The resulting lack of consensus on acceptability that developed among the physicists in our clinic prompted us to establish guidelines for action levels in the IMRT QA system. Two dosimetric parameters, dose difference and distance‐to‐agreement (DTA), are frequently used to evaluate the agreement between planned and delivered IMRT fields.^(13)^ According to some researchers, a 3% dose difference and 3 mm DTA in the planned and delivered IMRT fields constitute acceptable agreement between the two types of fields.^(14)^ Another widely used IMRT QA analysis tool, the gamma index (γ), also takes into account both dose difference and DTA.^(15)^ A γ of 1.0 or less indicates that a particular point falls within the 3% dose difference and 3 mm DTA criteria and, therefore, is an acceptable result. Although medical physicists continue to use these IMRT QA methods, definitive tolerance values for IMRT QA have yet to be established. In an effort to provide action levels for physicists working in our facility, we evaluated our initial experience with IMRT QA with portal dosimetry. We also established a strategy to improve our IMRT QA program. This paper describes our methods, providing a guideline for other facilities.

## II. MATERIALS AND METHODS

### A. Study design

In this study, we retrospectively reevaluated all 152 treatment plans, consisting of 1152 IMRT QA fields, delivered in our clinic between September 2006 and December 2006.

### B. Experiment facilities and software

Our clinic has four Varian linacs (two Trilogies and two 21EX linacs with Trilogy upgrades); all linacs are equipped with aS1000 flat‐panel imagers. The portal imager's support arm can be used to position the imager opposite the treatment head, allowing measurements of QA fields at the actual gantry angles that are used in a patient's treatment plan.

We used the ARIA integration system (version 8.0), the Eclipse treatment planning system, the Portal Vision System, and the Varian Portal Dosimetry System (all available from Varian Medical Systems) for portal dose prediction (PDP) calculations, treatment delivery, and data review.

### C. Portal dosimetry QA procedure

We performed IMRT QA with portal dosimetry for each treatment plan as follows:

- First, we calculated the PDP by superimposing the patients' treatment fields onto the portal imager's geometry at the designated SID using the Eclipse treatment planning system (Varian Medical Systems). We calculated a separate PDP for each field using the planned gantry angle, collimator rotation, field size, dynamic multileaf collimator (dMLC) sequence, dose rate, and number of monitor units (MU) as those to be used in delivering radiation to the patients. In instances in which a dimension of the treatment field exceeded the active area of the portal imager, we rotated the collimator by 90° to fit the entire field within the imager's active area. However, if this rotation is unsuccessful (e.g., there are still regions extending beyond the imager's active area), we recommend that IMRT QA be performed using other methods such as film and ion chambers.
- Second, we conducted an output check and then delivered the QA fields. To achieve the most accurate results, we recommend that an output check be conducted prior to performing IMRT QA. We delivered all IMRT treatments using a dMLC method, which is the standard treatment delivery technique used in our clinic. All QA fields were delivered to the patients prior to their third radiotherapy fraction with the actual machine used in their treatment. The measured EPID response was automatically converted to CU by reference to the calibration table for the specific linac, dose rate, and energy used in the treatment.
- Finally, we compared the EPID response with the PDP using the analysis software included in the portal dosimetry system.

### D. Analysis of portal dosimetric results

To evaluate the IMRT QA results with portal dosimetry, we used the gamma index values which are calculated for the irradiated fields over the entire active area of the portal imager. We evaluated the following three scalar parameters of agreement: maximum gamma $\left(\gamma_{\text{max}}\right)$, average gamma $\left(\gamma_{\text{avg}}\right)$, and percentage of the field area with a gamma value greater than $1.0\;(\gamma_{\%}>1)$; for all three variables, lower values indicate better agreement between the measured dose and the calculated PDP.

We documented the $\gamma_{\text{max}}$, $\gamma_{\text{avg}}$, and $\gamma_{\%}>1$ values for each treatment field and tabulated the mean values for each parameter and their associated standard deviations (SDs). These values were used to establish a set of action levels for our clinic. For each field, we also documented the number of MU, dose rate, number of control points (CP; which defines the number of dMLC segments per field in the treatment planning system), field type (“split” carriage or “non‐split” carriage), and treatment site. In addition, we calculated a parameter to estimate the average rate, number of control points delivered per minute (CP/min) at which field segments were delivered to determine if it had any effect on the agreement between the calculated PDP and the measured dose. For each treatment field, we determined the average number of control points delivered per minute (CP/min) by dividing the total number of CP by the MU divided by the dose rate (DR) [(CP/(MU/DR)].

### E. Method to improve agreement between calculated PDP and measured dose

To improve the agreement between the calculated PDP and the measured dose, we retrospectively reevaluated the 34 fields with the poorest agreement, that is, fields that had values for one or more parameters $\left(\gamma_{\text{max}},\;\gamma_{\text{avg}},\;\text{and}\;\gamma_{\%}>1\right)$, and $\gamma_{\%}>1)$ more than 3 SDs from the mean institutional value. Specifically, we changed the dose rate because we thought that it might be an important factor in determining the value of the gamma parameters. The majority of the fields in this study were delivered at a dose rate of 600 MU per minute, which is the dose rate commonly used in our clinic, but some fields were delivered at a lower dose rate (574±73; Table 1). In our clinic, the dose rate for each treatment plan is selected by the individual dosimetrist or medical physicist responsible for the treatment plan. Some physicists in our group noted a trend (undocumented) that QA results seemed better at lower dose rates than at higher ones. They suspected that the agreement between the MLC motion and MU specified for particular segments were not optimally synchronized at higher dose rates, thereby affecting the accuracy of the delivered plan. We therefore, replanned the treatments for the 34 cases with these “outlier” gamma parameters at lower dose rates and repeated the QA procedure for each new plan. In cases in which a dose rate of 600 MU per minute had been used, we lowered the dose rate to 400 MU per minute; in cases where the dose rate was 400 MU per minute, we lowered it to 300 MU per minute.

**Table 1:** Mean and standard deviations of agreement parameters for all fields investigated.^a^

*Analysis subcategory*	$\gamma_{\text{max}}$	$\gamma_{\text{avg}}$	$\gamma_{\%}>1$	*MU*	*DR*	*CP*	*CP/Min*
All fields (n=1152)	2.4±0.8	0.33±0.13	4.1±6.2	129±64	574±73	124±30	653±355
Split (n=472)	2.6±0.8	0.37±0.14	5.1±7.2	122±40	578±65	120±32	626±375
Non‐split (n=680)	2.3±0.7	0.32±0.13	3.5±5.3	133±74	570±77	126±28	672±353
Prostate (n=400)	2.3±0.7	0.27±0.10	1.6±2.7	120±35	596±26	129±27	677±201
H&N (n=397)	2.7±0.8	0.43±0.12	7.6±8.1	127±48	571±70	124±31	616±274
Brain (n=78)	2.3±0.8	0.36±0.09	4.5±4.3	94±25	593±62	142±26	956±305
Breast (n=67)	2.5±0.8	0.29±0.10	2.1±2.5	213±65	559±84	114±22	340±213
Breast SIB (n=20)	2.2±0.7	0.38±0.22	8.0±11.0	41±13	33±74	129±27	1181±582
Breast boost (n=24)	1.7±0.5	0.23±0.07	1.3±2.6	145±50	483±124	90±22	441±537
Ear or Eye (n=39)	2.1±0.8	0.35±0.06	3.8±3.6	100±40	536±94	97±12	570±181

^a^ Of these 1152 fields evaluated, 1025 were grouped into 7 treatment site categories. The remaining 127 fields could not be categorized into treatment sites, but are included in the all fields category.

## III. RESULTS AND DISCUSSION

**Figure 1:**
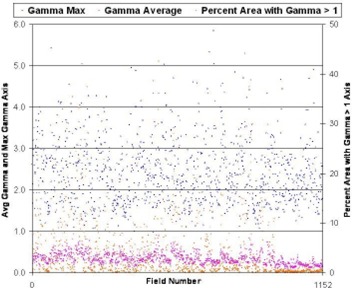
Scatter plot of $\gamma_{\text{max}}$, $\gamma_{\text{avg}}$, and $\gamma_{\%}>1$ for each field evaluated in this study. The institutional mean values for each parameter are shown on the right side of the plot with 1 SD and 2 SDs. Of the 1152 fields studied, 34 (3%) had one or more values greater than 3 SDs from the mean.

Fig. 1 shows a scatter plot of $\gamma_{\text{max}}$, $\gamma_{\text{avg}}$, and $\gamma_{\%}>1$ values for each of the 1152 treatment fields evaluated in our study. The scale of the $\gamma_{\text{max}}$ and $\gamma_{\text{avg}}$ values was approximately 10 × lower than that of the $\gamma_{\%}>1$ values. Thus, we plotted the $\gamma_{\text{max}}$ and $\gamma_{\text{avg}}$ values using the primary ordinate axis and the $\gamma_{\%}>1$ values using a secondary ordinate axis. Table 1 presents the mean values ±SD for each parameter for all fields and the mean ±SD based on the field type and treatment site. Of the 1152 fields studied, 92 (8%) had one or more values more than 2 SDs from the mean and 34 fields (3%) had one or more values greater than 3 SDs from the mean. Our data are in good agreement with data published by Zijtveld et al., who reported mean ±SD values of 0.43±0.13 for $\gamma_{\text{avg}}$ and of 6.1%±6.8% for γ>1 in their study of 75 patients.^(11)^

**Figure 2:**
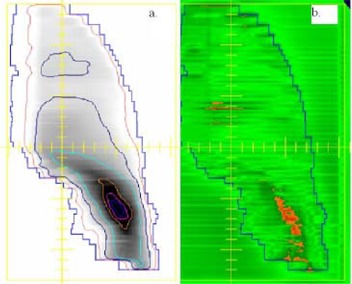
Portal dosimetry gamma evaluation showing good agreement between the measured dose and calculated PDP. (a) Overlay of measured image response and PDP. (b) Gamma map. Most of the field has gamma values less than or equal to 1.0 (green). The small area in the lower right quadrant of the field with gamma values greater than 1.0 (orange) corresponds to a high dose‐gradient region (shown in 2a).

**Figure 3:**
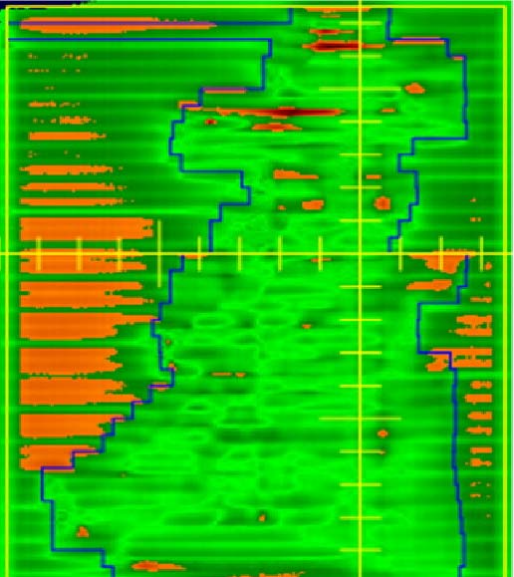
Portal dosimetry for a split field. As in areas that have gamma values greater than 1.0 are shown in orange. Most areas with a gamma value greater than 1.0 are located in regions where the dose was not modified by the MLC and also not shielded by the dimensions of the upper and lower jaws. Such regions would normally be expected to have low dose gradients, but in these regions, small differences in dose (due to, for example, MLC leakage) could cause large gamma values, as most likely occurred here.

**Figure 4:**
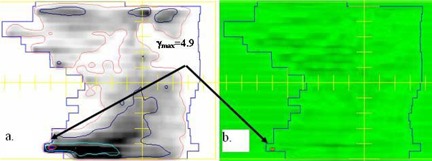
Portal dosimetry gamma evaluation for a split field with high $\gamma_{\text{max}}$ values. (a) Portal image with superimposed isodose lines from PDP. (b) Area with gamma values greater than 4.0 (orange). This shaded area corresponds to a high dose‐gradient region.

Figs. 2–4 show examples of the portal dosimetry evaluations. Although the viewer options in the portal dosimetry software allow the user to apply different settings in the presentation of gamma maps, we chose to use the default setting, which displays areas with gamma values less than or equal to 1.0 (agreement is within the 3%/3 mm) in green and areas with gamma values greater than 1.0 (agreement outside the 3%/3mm criteria) in a different color. Figs. 2 and 3 show examples of portal dosimetry gamma evaluations with the viewing scale set at 1.0. In Fig. 2, the gamma map shows that most of the field has gamma values less than or equal to 1.0 (large area shown in green; Fig. 2b); the small area in the lower right quadrant of the field has gamma values that are greater than 1.0, corresponding to a high dose‐gradient region (shown in orange; Fig. 2a). Because a majority of the field has gamma values less than or equal to 1.0 and areas with gamma values greater than 1.0 are confined to a high‐dose gradient region, there is good agreement between the calculated PDP and the measured dose. Fig. 3 shows a gamma map for a split‐field treatment. High gamma values were found in regions where the dose was not modified by the MLC and also not shielded by the dimensions of the upper and lower jaws. Such regions would normally be expected to have low dose gradients, but in such regions, small differences in dose (due to, e.g., MLC leakage) could cause large gamma values, as most likely occurred here. Fig. 4 shows a field that has a high $g_{\text{max}}\;\text{of}\;4.9$. In this figure, the viewing scale is shifted such that only areas with gamma values greater than 4.0 appear in the contrasting color (orange), allowing us to clearly show that these high $g_{\text{max}}$ values were confined to a very small area of the treatment field (Fig. 4b) and corresponded to a high dose‐gradient region (Fig. 4a).

### A. The effect of field type on agreement between calculated PDP and measured dose

We evaluated treatment fields that were delivered using multiple carriage positions (“split fields”) and a single carriage position (“non‐split fields”). We found that non‐split fields displayed slightly better agreement between the calculated PDP and measured dose than split fields (Table 1). Of the three gamma parameters that we evaluated, $\gamma_{\%}>1$ differed the most between the two field types; it was higher for the split fields (5.1%±7.2%) than for the non‐split fields (3.5%±5.3%).

Unlike non‐split fields, many split fields have large areas of the field receiving dose that is not modulated by the MLC and thus should have low dose gradients. We believe that most field areas with gamma values greater than 1.0 that were in such low dose‐gradient regions received additional dose primarily from interleaf MLC leakage and thus had higher $\gamma_{\%}>1$ values.

### B. The effect of treatment site on agreement between calculated PDP and measured dose

We also evaluated fields based on treatment site: head and neck, breasts, breast cavity boost, breast cavity simultaneous‐in‐field boost (SIB), prostate, brain, ears and eyes. Although we found that most of the parameters had similar mean values for the different treatment sites, we did notice some differences in the breast boost fields and head and neck fields.

Breast boost fields and breast SIB fields had the best and the worst agreement, respectively, between the calculated PDP and measured dose. There seems to be some correlation between CP/min and the agreement observed for these two treatment sites, which was 441±537 for the breast boost fields and 1181±582 for the breast SIB fields. These values indicate that, on average, the breast SIB fields delivered approximately 2.5×more CP/min than the breast boost fields, which means that the breast SIB fields had more modulation per unit time than the breast boost fields. This result was largely due to the design of the treatment plan. Both treatment plans consisted of two non‐coplanar fields, but the breast boost plan delivered 2.14 Gy per fraction, whereas the breast SIB plan delivered a lower dose of 0.34 Gy per fraction. The breast boost fields typically had a large number of MU (145±50) and a moderate level of dMLC segments (90±22 CPs). Conversely, the breast SIB fields typically had a lower number of MU (41±13) and a higher level of dMLC segments (129±27 CPs).

We also noted some differences for the head and neck treatment site. The mean value of the $\gamma_{\%}>1$ parameter (7.6%±8.1%) was higher for the head and neck treatment site than all other treatment sites (excluding the breast SIB). Most (69%) head and neck fields were split fields. Thus, the high $\gamma_{\%}>1$ parameters were largely due to dose leakage into the low dose‐gradient regions, as described in the previous section.

### C. Clinical action levels

We set action levels in our clinic using the gamma values calculated here. Specifically, we set action levels based on the institutional mean values for all fields and their standard deviations (Table 2). When establishing the clinical action levels, we excluded the values for treatment field type and site because having too many reference values would make applying the clinical action levels exceedingly complex. Our objective was to simplify the analysis, not to further complicate the process. Clinical action levels were established as follows:

**Table 2:** Institutional mean values for $\gamma_{\text{max}},\;\gamma_{\text{avg}},\;\text{and}\;\gamma_{\%}>1$.

	$\gamma_{\text{max}}$			$\gamma_{\text{avg}}$			$\gamma_{\%}>1$	
Mean	+1SD	+2SD	Mean	+1SD	+2SD	Mean	+1SD	+2SD
2.42	3.20	3.98	0.34	0.47	0.61	4.1	10.4	16.6

1. The average values for $\gamma_{\text{max}}$, $\gamma_{\text{avg}}$, and $\gamma_{\%}>1$ or all fields in a given treatment plan must be within 1 SD of the institutional mean values.
2. No more than 25% of the fields in a given treatment plan can have $\gamma_{\text{max}}$, $\gamma_{\text{avg}}$, or $\gamma_{\%}>1$ values greater than 2 SDs from the institutional mean values.

In instances when a plan does meet these action level criteria, the deviation is still considered acceptable if it meets one of the following allowed exceptions:

1. The average value for $\gamma_{\%}>1$ for all fields in a given treatment plan can be up to 2 SDs from the institutional mean value if the medical physicist is confident that the majority of the areas with values greater than 1.0 are outside the modulated field, e.g., in a low dose‐gradient region.
2. Clinical action level 2 above can be disregarded for the $\gamma_{\text{max}}$ parameter only if the areas (in disagreement) are very small and located in a high dose‐gradient region of the field.

In general, we rely on the experience of the clinical medical physicist to determine if a particular field or plan not meeting the criteria is acceptable. Portal dosimetry has a line profile evaluation tool which the medical physicist can use to perform additional evaluations. Because this tool allows profiles to be drawn in the x‐y plane and specify dose difference and DTA along the drawn axis, it can be an especially useful tool for evaluating a region of concern in a particular treatment field.

If the plan fails to fall within the clinical action levels, does not meet one of the allowed exceptions, and the medical physicist determines that the IMRT QA result is unacceptable, IMRT QA should be repeated using the strategy of lowering the dose rate (described in the following section). However, we must emphasize that simply changing the dose rate in the record and verify system is insufficient; the leaf motions and dose should also be recalculated at the new “lower” dose rate in the treatment planning system. The number of control points for each field is specifically optimized for the assigned dose rate by the leaf motion calculator in the treatment planning system at the time of the calculation. If the strategy of lowering the dose rate fails to improve agreement and the nature of the disagreement can not be acceptably resolved, an entirely new treatment plan should be developed.

### D. The effect of dose rate on agreement between calculated PDP and measured dose

We found that recalculating the fields at lower dose rates was an effective strategy for decreasing gamma values, thereby improving the agreement between calculated PDP and measured dose for fields with significant modulation and/or low numbers of MU. Using this strategy, we were able to improve the agreement between calculated PDP and measured dose in 30 of the 34 fields that we replanned. In 4 fields, we found that lowering the dose rate actually resulted in a worse agreement between the calculated PDP and measured dose than that found with the initial higher dose rate. Table 3 provides the mean values ±SDs for $\gamma_{\text{max}}$, $\gamma_{\text{avg}}$, $\gamma_{\%}>1$, MU/DR, and CP/min for fields that were replanned with lower dose rates. Prior to replanning, the average CP/min for these fields was 552±281. After we lowered the dose rate, the average number of CP/min decreased to 368±193. Taken together, our findings suggest that lowering the dose rate can be an effective strategy for improving agreement between the calculated PDP and the measured dose. This improvement may be linked to an increase in the time allotted for the delivery of each field segment (low number of CP/min).

**Table 3:** Mean and standard deviations of agreement parameters, MU/DR, and CP/min for the 34 fields that had one or more values $\left(\gamma_{\text{max}},\;\gamma_{\text{avg}},\;\gamma_{\%}>1\right)$ greater than 3 SDs from the mean which were replanned at lower dose rates.^a^

*Dose rate evaluation time (fields)*	$\gamma_{\text{max}}$	$\gamma_{\text{avg}}$	$\gamma_{\%}>1$	*MU/DR*	*CP/min*
Before (all 34 original fields)	3.97±0.84	0.51±0.19	14.6±11.1	0.35±0.24	552±281
After (all 34 replanned fields)	3.45±0.70	0.39±0.15	8.5±9.7	0.48±0.37	368±193
After (30 improved fields)	3.37±0.70	0.38±0.14	8.0±8.9	0.50±0.39	367±196

^a^ Data are shown for the following conditions: original data with no replanning, all replanned data (34 fields), and replanned data for 30 fields where lowering the dose rate was effective in improving the agreement between the PDP and measured dose lower dose.

Because this strategy was not successful in all 34 fields that were replanned, we strongly recommend that when a new plan is recalculated at a lower dose rate, the new plan IMRT QA be completed within 24 h of the original QA. Furthermore, the new plan should be used to treat a patient only if it improves IMRT QA results.

### E. Reduction in documentation

**Figure 5:**
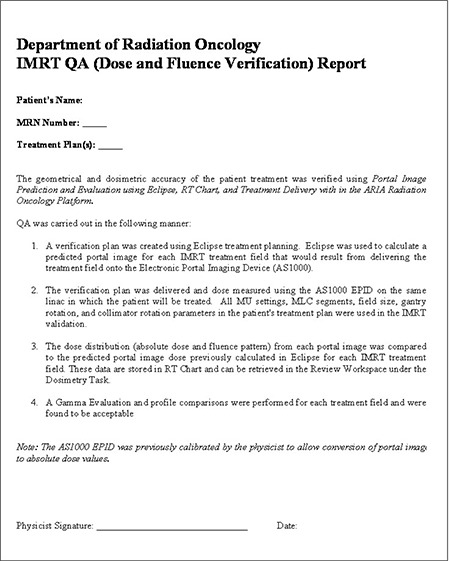
IMRT QA report template. This document is attached to the electronic treatment record for each patient after IMRT QA is completed. The document is also printed and signed by the medical physicist who completed the QA and is added to the patient's paper chart.

We found that the implementation of IMRT QA with portal dosimetry greatly reduced the amount of IMRT paperwork that needs to be filed in a patient's chart. Fig. 5 shows the only IMRT document filed in a patient's paper chart in our clinic. This document describes the QA procedure, shows that the results were acceptable, includes the date that the QA was completed, and notes that each QA field portal image is stored in the database as part of the patient's electronic record. This document is also added to the patient's electronic record using the ARIA integration system.

## V. CONCLUSIONS

Using our findings, we set clinical action levels for performing IMRT QA with portal dosimetry in our clinic. We also provided a valuable strategy that medical physicists can use to improve the results of IMRT QA for individual patients. We must caution that these clinical action levels are not intended to replace the medical physicist's clinical experience. In fact, medical physicists must carefully evaluate IMRT fields that are not within the clinical action levels to ascertain the causes for the disagreement and the suitability of these fields for patient treatment. We conclude that these clinical action levels are a useful tool for standardizing the evaluation of EPID‐based IMRT QA.
